# IFN‐γ and IL‐17A Exhibit Opposing Roles in Age‐Related Blood Pressure Dysregulation

**DOI:** 10.1155/ijhy/8370291

**Published:** 2026-04-27

**Authors:** Lweendo Muchaili, Elletina Nchimunya Sinamwenda, Benson M. Hamooya, Joreen P. Povia, Annet Kirabo, Sepiso K. Masenga

**Affiliations:** ^1^ Pathology and Microbiology Department, School of Medicine and Health Sciences, Mulungushi University, Livingstone, Zambia, mu.ac.zm; ^2^ Nursing Department, School of Nursing and Midwifery, Mulungushi University, Kabwe, Zambia, mu.ac.zm; ^3^ Department of Cardiovascular Science and Metabolic Diseases, Livingstone Center for Prevention and Translational Sciences, Livingstone, Zambia; ^4^ Department of Biostatistics and Public Health, Livingstone Center for Prevention and Translational Sciences, Livingstone, Zambia; ^5^ Department of Public Health, School of Medicine and Health Sciences, Mulungushi University, Livingstone, Zambia, mu.ac.zm; ^6^ Department of Medicine, Vanderbilt University Medical Center, Nashville, Tennessee, USA, vanderbilt.edu; ^7^ Vanderbilt Center for Immunobiology, Vanderbilt Institute for Infection, Immunology and Inflammation, Vanderbilt University Medical Center, Nashville, Tennessee, USA, vanderbilt.edu; ^8^ Vanderbilt Institute for Global Health, Vanderbilt University Medical Center, Nashville, Tennessee, USA, vanderbilt.edu

**Keywords:** age, BMI, HIV, inflammaging, inflammation, interferon-gamma, interleukin-17, systolic blood pressure

## Abstract

**Background:**

The inflammatory pathophysiology of hypertension involves interactions between innate and adaptive immune pathways. While interleukin‐6 (IL‐6) and tumor necrosis factor‐alpha (TNF‐α) have established roles, the contributions of specific T cell–derived cytokines, especially interferon‐γ (IFN‐γ), interleukin‐17A (IL‐17A), and interleukin‐1 (IL‐1), remain less defined in age‐stratified human populations. The aging immune system undergoes characteristic shifts that may differentially engage these pathways in regulating blood pressure, with this immune senescence being notably accelerated in people living with HIV (PLWH). Therefore, this study aimed to investigate age‐stratified associations between key cytokines and systolic blood pressure (SBP) and to determine how these relationships interact with metabolic factors in both PLWH and non‐PLWH.

**Methods:**

We conducted a cross‐sectional analysis of 332 adults, stratified into two age groups: < 45 years (*n* = 121) and ≥ 45 years (*n* = 211). Of the participants, 241 were living with HIV (PLWH) and 91 were HIV‐negative. Multiple linear regression models, adjusted for demographics, HIV status, LDL cholesterol, and smoking, assessed associations between SBP and plasma levels of IFN‐γ, IL‐17A, IL‐6, IL‐1, and TNF‐α. Analyses were performed separately for each age group. In secondary analysis, we evaluated the cytokine–SBP associations using multilinear regression within the HIV + subgroup and in a pooled model including both PLWH and HIV‐negative participants to assess consistency across HIV serostatus.

**Results:**

The older cohort had significantly higher median SBP, BMI, and LDL cholesterol (all *p* < 0.001). Regression models revealed distinct, age‐dependent drivers of SBP. In adults < 45 years, only IFN‐γ (*β* = 0.00107, *p* = 0.018) and chronological age (*β* = 0.487, *p* = 0.047) showed significant positive associations with SBP. In contrast, for adults ≥ 45 years, body mass index (BMI) was the dominant and consistent predictor across all models (*β* range: 1.95–2.08, all *p* < 0.001). In this older group, IL‐17A was additionally significant in its specific model (*β* = 0.00322, *p* = 0.0448). Systemic levels of IL‐6, IL‐1, and TNF‐α were not significantly associated with SBP in either cohort. Secondary analysis showed consistent cytokine–SBP relationships by HIV status, with a slight reduction in effect size seen in IL‐17A after adding HIV‐negative participants: HIV+ (*β* = 0.0029, *p* = 0.035) to the combined cohort (*β* = 0.0027, *p* = 0.048). IFN‐γ remained stable with near‐identical effect sizes (HIV+: *β* = 0.0010, *p* = 0.011; combined: *β* = 0.0010, *p* = 0.010).

**Conclusion:**

The regulation of blood pressure appears to undergo a fundamental pathophysiological transition with advancing age. In younger adults (< 45 years), SBP is associated with adaptive immune activity, mainly the Th1‐associated IFN‐γ. After midlife (≥ 45 years), this relationship is superseded by a model dominated by adiposity, within which the Th17‐associated IL‐17A acts as a contributing inflammatory mediator. These age‐stratified cytokine–blood pressure relationships were consistent across PLWH and HIV‐negative individuals, suggesting that this pathophysiological succession operates independently of HIV serostatus. Collectively, these findings advocate for age‐tailored, mechanism‐based approaches to the understanding and managing blood pressure–related risk.

## 1. Introduction

The age‐related​ rise in systolic blood pressure (SBP) is mechanistically multifactorial, involving vascular, renal, and neurohormonal pathways [1, 2]. Traditional risk factors offer only a partial explanation, whereas the role of age‐related immune dysregulation, often termed inflammaging, provides a more compelling pathophysiological framework. Inflammaging is a state of chronic, low‐grade systemic inflammation, which is characterized by a dysregulated cytokine environment that progressively impairs vascular homeostasis [3]. Although extensive research has linked various innate immune mediators including interleukin‐6 (IL‐6), tumor necrosis factor‐alpha (TNF‐α), and C‐reactive protein (CRP) to elevated blood pressure (BP), this perspective likely represents an incomplete picture of immune, cardiovascular interactions. The contributions of adaptive immune pathways, especially the interferon and interleukin‐17 families, remain underexplored in human hypertension, despite their established roles in vascular biology and sterile inflammation [4]. Interferon‐gamma (IFN‐γ), the prototypical cytokine of T‐helper 1 (Th1) lymphocytes, is a potent activator of macrophages and endothelial cells, capable of driving vascular inflammation and endothelial dysfunction and promoting hypertensive vascular remodeling in experimental models. In parallel, interleukin‐17A (IL‐17A), the hallmark cytokine of Th17 lymphocytes, stimulates the production of other pro‐inflammatory cytokines and chemokines, recruits neutrophils, and has been implicated in various autoimmune conditions and in atherosclerotic plaque instability. These cytokines represent distinct immune pathways with potentially unique contributions to cardiovascular pathophysiology [5, 6].

Inflammaging and immune remodeling are of great relevance in conditions characterized by chronic immune activation, such as HIV infection. People living with HIV (PLWH) experience accelerated immune senescence, persistent low‐grade inflammation, and heightened cardiovascular risk, even with effective antiretroviral therapy [7, 8]. Thus, PLWH are likely to have an exaggerated form of the age‐dependent relationship between immune dysregulation and BP.

Crucially, the activity of these specialized immune pathways is not static across the human lifespan but rather shaped by the process of immune senescence. The aging immune system undergoes characteristic shifts, including thymic involution, expansion of memory T‐cell pools, and altered cytokine production profiles. This immunologic remodeling likely creates an environment where specific inflammatory pathways become preferentially engaged in the pathogenesis of age‐related conditions, such as hypertension. Furthermore, the relationship between systemic inflammation and BP may be modulated by the evolving dominance of metabolic factors, such as adiposity, which itself exhibits a strong age‐dependent association with hypertension [9–11].

We therefore posit that the relationship between specific immune signatures and BP is age‐dependent. Rather than exhibiting uniform associations across adulthood, different inflammatory mediators may become relevant at distinct life stages, reflecting an evolving pathophysiology. This study, therefore, tests the hypothesis that circulating levels of key cytokines representing major immune axes, including IFN‐γ, IL‐17A, IL‐6, TNF‐α, and IL‐1, demonstrate age‐stratified associations with SBP in both PLWH and those without HIV. We further hypothesize that in older adults, these inflammatory associations may be superseded or mediated by the dominant influence of metabolic factors, such as body mass index (BMI), showing a critical transition in the primary drivers of hypertension across the lifespan. Through stratified analyses, we aim to clarify these age‐specific relationships and advance a life‐course understanding of how inflammation influences BP regulation.

## 2. Methods

### 2.1. Study Design and Participants

This cross‐sectional analysis included 332 (241 PLWH, 91 HIV‐negative) adults recruited from a tertiary hospital in Livingstone, Zambia. Participants were drawn from a larger parent cohort of individuals with known SBP status who attended routine medical checkups and had been previously characterized in an earlier dietary intervention study. PLWH constituted a substantial proportion of this parent cohort and thus influenced the composition of the current analysis. This composition, however, enabled investigation of cytokine–BP relationships in a population with heightened chronic inflammation, while also permitting comparison of these relationships across HIV status groups [12, 13]. Eligible participants were aged ≥ 18 years. Key exclusion criteria included diabetes, pregnancy, active infections, and severe renal or hepatic disease to minimize confounding influences on inflammatory markers.

### 2.2. BP and Hypertension Definition

Resting BP was measured three times in a seated position using an automated Omron HEM‐7120 monitor, and the mean was used for analysis. Hypertension was defined as SBP ≥ 140 mmHg, diastolic BP ≥ 90 mmHg, or current use of antihypertensive medication.

### 2.3. Cytokine and Inflammatory Biomarker Quantification

Fasting blood samples were collected. Plasma cytokine concentrations (TNF‐α, IL‐17A, IFN‐γ, IL‐6, and IL‐1) were measured using commercially available enzyme‐linked immunosorbent assay (ELISA) kits according to the manufacturer’s protocols (Elabscience Biotechnology Co., Ltd., Houston, TX, USA). These kits are intended for research use only (RUO) and are not validated for clinical diagnostic purposes. Samples were diluted 100‐fold as recommended by the manufacturer to optimize assay performance within the dynamic range of the standard curve, and standard curves were generated using the provided standards. Cytokine concentrations were calculated from the standard curves using four‐parameter logistic regression, and final values were adjusted for the dilution factor.

### 2.4. Statistical Analysis

Participants were stratified into two age groups for the primary analysis: younger (< 45 years) and older (≥ 45 years). Continuous variables are presented as median (interquartile range) and were compared using *t*‐tests or Mann–Whitney *U* tests, as appropriate. Categorical variables were compared using chi‐square tests.

The associations between cytokine levels (IFN‐λ1, IFN‐γ, and IL‐17A) and SBP were assessed using multivariable linear regression models, adjusted for age, sex, HIV status, and BMI. These models were constructed separately for the younger and older age strata to test the primary hypothesis of age‐dependent associations. A two‐tailed *p* value < 0.05 was considered statistically significant. Secondary analyses were performed to assess whether the association between cytokines (IFN‐γ and IL‐17A) and SBP differed by HIV status. In these secondary analyses, the sample was stratified by HIV status, and the regression models were repeated within each subgroup to determine whether the association between cytokines and SBP differed between PLWH and HIV‐negative participants. All analyses were performed using StatCrunch.

### 2.5. Ethical Considerations

The study protocol received ethical approval from the Mulungushi University School of Medicine and Health Sciences Research Ethics Committee (MUHSREC; Ref. No.: SMHS‐MU3‐2023‐005) and the Livingstone University Teaching Hospital. Written informed consent was obtained from all participants after a comprehensive verbal explanation of the study objectives, procedures, risks, and benefits in a language of their understanding. Participation was voluntary, and data confidentiality was strictly maintained throughout the research.

## 3. Results

### 3.1. Basic Description of the Study Populations

The study included 332 participants, divided into two age strata: 121 individuals younger than 45 years (median age 35.5 years) and 211 individuals aged 45 years or older (median age 56 years). Of the total cohort, 241 participants were living with HIV, while 91 were HIV‐negative. A comparison of the groups revealed significant differences in key cardiometabolic and demographic factors, but not in sex distribution or most inflammatory markers.

The older cohort (≥ 45 years) exhibited a markedly worse cardiometabolic risk profile. They had significantly higher median SBP (131 vs. 116 mmHg, *p* < 0.0001), BMI (25.48 vs. 22.62 kg/m^2^, *p* = 0.0008), and LDL cholesterol levels (3.26 vs. 2.57 mmol/L, *p* < 0.0001). Furthermore, the prevalence of HIV was significantly greater in the older group (78.3% vs. 65.4%, *p* = 0.007). The proportion of current smokers was low in both groups and not statistically different (5.6% in older vs. 10.7% in younger, *p* = 0.093). The sex distribution was similar, with a male predominance of approximately 70% in both groups (*p* = 0.40).

Notably, despite the significant differences in clinical parameters, the circulating levels of key pro‐inflammatory cytokines, including TNF‐α, IL‐17A, IFN‐γ, IL‐6, and IL‐1, did not differ significantly between the younger and older participants. This suggests that the substantial age‐related increase in SBP and other risk factors may not be directly attributable to gross differences in the systemic concentrations of these specific inflammatory mediators, pointing instead to other mechanisms, such as metabolic dysregulation, vascular aging, or tissue‐specific inflammation (Table 1).

**TABLE 1:** Demographic, clinical, and immunometabolic characteristics of the study participants by age group.

Characteristics	< 45 years (*n* = 121)	≥ 45 years (*n* = 211)	*p* value
Demographics			
Age (years), median (IQR)	35.5 (15)	56 (13)	**< 0.0001**
Sex, *n* (%) male	99 (72.8)	158 (68.7)	0.40
Anthropometrics			
BMI (kg/m^2^), median (IQR)	22.62 (7.35)	25.48 (8.16)	**0.0008**
Cardiometabolic and risk factors			
Systolic BP (SBP), median (IQR)	116 (20)	131 (28)	**< 0.0001**
LDL cholesterol (mmol/L), median (IQR)	2.57 (1.17)	3.26 (1.34)	**< 0.0001**
Smoking status, *n* (%) smoker	13 (10.7)	12 (5.6)	0.093
HIV status, *n* (%) positive	78 (64.5)	163 (78.3)	**0.007**
Inflammatory and immunometabolic markers, median (IQR)			
TNF‐α (pg/mL)	6.98 (30.40)	5.49 (40.94)	0.965
IL‐17A (pg/mL)	22.17 (105.2)	22.90 (660.5)	0.312
IFN‐γ (pg/mL)	5.40 (22.07)	17.55 (23.10)	0.159
IL‐6 (pg/mL)	1.23 (100.4)	93.65 (107.9)	0.091
IL‐1 (pg/mL)	3.87 (88.32)	4.79 (251.5)	0.204

*Note:* IQR, interquartile range; IFN‐γ, interferon‐gamma; IL, interleukin. Bold *p* values indicate statistical significance (*p* < 0.05).

Abbreviations: BMI, body mass index; LDL, low‐density lipoprotein; SBP, systolic blood pressure; TNF‐α, tumor necrosis factor‐α.

### 3.2. Regression Analysis Stratified by Age Group

To investigate age‐specific determinants of SBP, multiple linear regression analyses were conducted separately for participants under 45 years and those 45 years or older. The models adjusted for core demographic and clinical variables (age, BMI, HIV status, LDL cholesterol, sex, and smoking status) while sequentially incorporating specific immunological markers. The results, highlighting distinct age‐related associations, are presented below.

### 3.3. Association of IFN‐γ With SBP by Age

A model examining IFN‐γ showed differing factors across age groups (Table 2). In the older cohort (≥ 45 years), BMI was the sole significant associate, showing a strong positive association with SBP (*β* = 1.95, *p* = 0.0005). In contrast, within the younger cohort (< 45 years), both chronological age (*β* = 0.49, *p* = 0.0475) and higher circulating levels of IFN‐γ (*β* = 0.00107, *p* = 0.018) were independently associated with increased SBP.

**TABLE 2:** Multiple linear regression for systolic blood pressure with IFN‐γ, stratified by age group.

Variable	< 45			≥ 45		
	*Β*	95% CI	*p* value	*Β*	95% CI	*p* value
Age at visit	0.48	0.00–0.97	0.047	−0.047	−0.68–0.58	0.88
BMI	0.68	−0.02–1.39	0.058	1.95	0.90–3.00	**0.0005**
HIV status	1.81	−6.45–10.08	0.66	2.52	−10.70–15.75	0.70
LDL cholesterol	1.92	−1.92–5.77	0.32	3.63	−2.48–9.75	0.23
Sex (male)	−4.058	−12.69–4.57	0.35	−6.52	−17.25–4.20	0.22
Smoking status	−8.73	−26.28–8.81	0.32	−2.67	−25.47–20.11	0.81
IFN‐γ	0.0010	0.0002–0.0020	**0.018**	−0.0018	−0.0067–0.0029	0.44

*Note:* IFN‐γ, interferon‐gamma; sex reference is female. Bold *p* values indicate statistical significance (*p* < 0.05).

Abbreviations: BMI, body mass index; CI, confidence interval; LDL, low‐density lipoprotein.

### 3.4. Association of IL‐17A With SBP by Age

The model incorporating IL‐17A showed a different pattern of age‐specific associations (Table 3). For individuals aged 45 and above, both higher BMI (*β* = 2.00, *p* = 0.0003) and elevated IL‐17A levels (*β* = 0.0032, *p* = 0.044) were significantly associated with increased SBP.

**TABLE 3:** Multiple linear regression for systolic blood pressure with IL‐17A, stratified by age group.

Variable	< 45			≥ 45		
	*Β*	95% CI	*p* value	*Β*	95% CI	*p* value
Age at visit	0.460	−0.03–0.95	0.0659	−0.037	−0.65–0.58	0.904
BMI	0.683	−0.04–1.40	0.0626	1.999	0.97–3.03	0.0003
HIV status	2.340	−6.33–11.01	0.594	−4.416	−15.33–6.50	0.421
LDL cholesterol	2.028	−1.91–5.97	0.311	1.694	−4.39–7.78	0.579
Sex (male)	−3.651	−12.51–5.21	0.416	−3.406	−14.15–7.34	0.528
Smoking status	−9.049	−26.95–8.85	0.319	0.316	−26.40–27.03	0.981
IL‐17A	0.00258	−0.0026–0.0078	0.331	0.00322	0.0001–0.0064	0.044

*Note:* IL‐17A, interleukin 17A; sex reference is female.

Abbreviations: BMI, body mass index; CI, confidence interval; LDL, low‐density lipoprotein.

### 3.5. Association of IL‐6 With SBP by Age

The analysis examining IL‐6 demonstrated a consistent pattern of age‐specific risk factors (Table 4). For participants aged 45 and above, BMI remained the only significant associate of SBP (*β* = 2.08, *p* = 0.0011). In contrast, within the younger cohort (< 45 years), increasing age (*β* = 0.44, *p* = 0.083) was the primary factor showing association, while IL‐6 levels showed a nonsignificant positive trend (*β* = 0.103, *p* = 0.11).

**TABLE 4:** Multiple linear regression for systolic blood pressure with IL‐6, stratified by age group.

Variable	< 45			≥ 45		
	*Β*	95% CI	*p* value	*Β*	95% CI	*p* value
Age at visit	0.43	−0.05–0.93	0.0806	−0.053	−0.78–0.67	0.88
BMI	0.66	−0.05–1.38	0.0674	2.07	0.87–3.28	0.0011
HIV status	1.39	−7.52–10.31	0.757	−10.05	−27.72–7.61	0.25
LDL cholesterol	2.05	−1.86–5.98	0.300	3.42	−3.57–10.41	0.32
Sex (male)	−3.69	−12.50–5.10	0.407	−4.31	−15.93–7.29	0.45
Smoking status	−10.9	−28.98–7.06	0.231	−1.43	−29.22–26.34	0.91
IL‐6	0.011	−0.0062–0.029	0.198	0.103	−0.027–0.23	0.11

*Note:* IL‐6, interleukin 6; sex reference is female.

Abbreviations: BMI, body mass index; CI, confidence interval; LDL, low‐density lipoprotein.

### 3.6. Association of IL‐1 With SBP by Age

Similar to other cytokines, the model incorporating IL‐1 showed differential associations by age (Table 5). For the older group (≥ 45 years), higher BMI was strongly and significantly associated with increased SBP (*β* = 1.99, *p* = 0.0004). No other variables, including IL‐1 levels, reached significance in this cohort. For the younger group (< 45 years), the pattern mirrored previous findings: Age itself was the primary variable showing an association (*β* = 0.44, *p* = 0.081), while IL‐1 showed no significant link (*β* = 0.012, *p* = 0.198).

**TABLE 5:** Multiple linear regression for systolic blood pressure with IL‐1, stratified by age group.

Variable	< 45			≥ 45		
	*Β*	95% CI	*p* value	*Β*	95% CI	*p* value
Age at visit	0.43	−0.05–0.93	0.080	−0.044	−0.68–0.59	0.88
BMI	0.66	−0.05–1.38	0.067	1.98	0.94–3.04	0.0004
HIV status	1.39	−7.52–10.31	0.75	−2.55	−14.40–9.28	0.66
LDL cholesterol	2.05	−1.86–5.98	0.30	2.56	−3.57–8.70	0.40
Sex (male)	−3.69	−12.50–5.10	0.40	−4.44	−16.02–7.14	0.44
Smoking status	−10.96	−28.98–7.06	0.23	−1.34	−24.25–21.57	0.90
IL‐1	0.011	−0.0062–0.029	0.19	0.0036	−0.0065–0.013	0.46

*Note:* IL‐1, interleukin 1; sex reference is female.

Abbreviations: BMI, body mass index; CI, confidence interval; LDL, low‐density lipoprotein.

### 3.7. Association of TNF‐α With SBP by Age

The final model evaluated TNF‐α, another major inflammatory mediator (Table 6). In the older cohort (≥ 45 years), BMI was yet again a significant associate of SBP (*β* = 2.00, *p* = 0.0007). TNF‐α levels showed no significant association in either age group. For younger participants, the model showed no significant associations, though age (*β* = 0.46, *p* = 0.065) and BMI (*β* = 0.72, *p* = 0.055) exhibited strong trends.

**TABLE 6:** Multiple linear regression for systolic blood pressure with TNF‐α, stratified by age group.

Variable	< 45			≥ 45		
	Β	95% CI	*p* value	Β	95% CI	*p* value
Age at visit	0.46	−0.03–0.96	0.064	−0.068	−0.71–0.58	0.83
BMI	0.71	−0.01–1.45	0.054	2.001	0.89–3.11	0.0007
HIV status	2.95	−5.46–11.37	0.48	−0.69	−11.31–9.93	0.89
LDL cholesterol	2.15	−1.80–6.11	0.28	2.98	−3.11–9.09	0.33
Sex (male)	−2.92	−11.75–5.89	0.51	−6.058	−16.77–4.66	0.26
Smoking status	−8.64	−26.78–9.49	0.34	−2.28	−25.21–20.64	0.84
TNF‐α	0.000067	−0.00043–0.00056	0.78	0.00011	−0.00106–0.00129	0.84

*Note:* Sex reference is female.

Abbreviations: BMI, body mass index; CI, confidence interval; LDL, low‐density lipoprotein; TNF‐α, tumor necrosis factor α.

### 3.8. Secondary Analyses

#### 3.8.1. Association of IL‐17A With SBP by HIV Status

Given​ the differing age‐specific patterns observed for IL‐17A and IFN‐γ, we performed secondary analyses to assess whether HIV status influenced these cytokine and SBP associations. Separate multiple linear regression models were constructed for the HIV + subgroup and the combined cohort to compare effect sizes and determine the robustness of these relationships across HIV serostatus.

#### 3.8.2. Association of IL‐17A and IFN‐γ With SBP by HIV Status

In the HIV + subgroup, in multiple linear regression model, IL‐17A was significantly associated with increased SBP (*β* = 0.0029, 95% CI: 0.0002–0.0057, *p* = 0.035), alongside age (*β* = 0.610, *p* = 0.0004) and BMI (*β* = 1.169, *p* = 0.0011).

When the HIV‐negative participants were added to the model, IL‐17A remained significantly associated with SBP, though the effect size was slightly attenuated (*β* = 0.0027, 95% CI: 0.00003–0.0054, *p* = 0.048). Age and BMI also remained significant associates of SBP (Table 7).

**TABLE 7:** Multiple linear regression for systolic blood pressure with IL‐17A, HIV + subgroup vs. combined cohort.

Variable	HIV + subgroup			Combined cohort		
	*β*	95% CI	*p* value	*β*	95% CI	*p* value
Age at visit	0.61	0.28–0.94	0.0004	0.41	0.18–0.63	0.0005
BMI	1.16	0.48–1.86	0.0011	0.94	0.37–1.50	0.0013
Sex (male)	−3.25	−11.44–4.94	0.44	−5.04	−11.28–1.20	0.11
Smoking status	−5.43	−19.25–8.38	0.44	−7.60	−21.54–6.34	0.29
LDL cholesterol	1.23	−2.46–4.94	0.51	2.25	−0.88–5.38	0.16
IL‐17A	0.0029	0.0002–0.0057	0.035	0.0027	0.00003–0.0054	0.048

*Note:* IL‐17A, interleukin 17A; sex reference is female.

Abbreviations: BMI, body mass index; CI, confidence interval; LDL, low‐density lipoprotein.

#### 3.8.3. Association of IFN‐γ With SBP by HIV Status

In the HIV + subgroup, IFN‐γ had a significant positive association with SBP (*β* = 0.00103, 95% CI: 0.0002–0.0018, *p* = 0.011), alongside age (*β* = 0.57, *p* = 0.0008) and BMI (*β* = 1.19, *p* = 0.0008).

When HIV‐negative participants were added to the model, IFN‐γ remained significantly associated with SBP, with a nearly identical effect size (*β* = 0.0010, 95% CI: 0.00025–0.0018, *p* = 0.010). Age and BMI also remained significant associates. See Table 8 below.

**TABLE 8:** Multiple linear regression for systolic blood pressure with IFN‐γ, HIV + subgroup vs. combined cohort.

Variable	HIV + subgroup			Combined cohort		
	*β*	95% CI	*p* value	*β*	95% CI	*p* value
Age at visit	0.57	0.24–0.90	0.0008	0.39	0.16–0.61	0.0009
BMI	1.19	0.51–1.87	0.0008	0.94	0.38–1.50	0.0011
Sex (male)	−4.77	−12.80–3.26	0.24	−5.76	−11.93–0.41	0.067
Smoking status	−5.56	−18.46–7.34	0.39	−7.73	−20.78–5.33	0.24
LDL cholesterol	1.51	−2.06–5.09	0.40	2.36	−0.71–5.43	0.13
IFN‐γ	0.0010	0.0002–0.0018	0.011	0.0010	0.00025–0.0018	**0.010**

*Note:* IFN‐γ, interferon‐gamma; sex reference is female. Bold *p* values indicate statistical significance (*p* < 0.05).

Abbreviations: BMI, body mass index; CI, confidence interval; LDL, low‐density lipoprotein.

## 4. Discussion

The findings of this study contribute to an improved, age‐stratified perspective on the literature regarding inflammation and hypertension, challenging the prevailing notion that a linear, cumulative inflammatory burden drives age‐related increases in BP [14]. A key observation is the dissociation between systemic cytokine levels and SBP across age groups. While the older cohort had statistically significantly higher SBP, LDL cholesterol, and BMI, the circulating concentrations of major pro‐inflammatory cytokines, TNF‐α, IL‐17A, IFN‐γ, IL‐6, and IL‐1, were not significantly elevated compared to younger participants. This stands in contrast to numerous epidemiological studies that report a positive correlation between markers with both age and hypertension [4, 15]. Our data suggest that the gross systemic concentration of these mediators may be a less sensitive biomarker for the specific inflammatory processes that directly elevate vascular resistance. This aligns with emerging understanding of these phenomena: that localized, tissue‐specific inflammation, within the vascular adventitia, perivascular adipose tissue, kidney, or sympathetic nervous system, is the critical driver, often operating independently of systemic levels [16–18]. Our negative findings for systemic cytokines, therefore, do not refute the inflammatory hypothesis of hypertension but rather refine it, directing focus away from the plasma proteome and toward the spatial biology of inflammatory signaling in target organs.

### 4.1. A Model of Pathophysiological Succession

The age‐stratified regression models showed a pathophysiological shift, offering a potential resolution to conflicting reports in the literature about the relative importance of immune versus metabolic factors. In participants under 45, increasing chronological age and higher levels of the Th‐1‐associated IFN‐γ were significant independent associates of SBP. This association with Th1‐driven immunity in younger adults finds support in experimental models where IFN‐γ contributes to endothelial dysfunction and vascular remodeling, and it suggests that adaptive immune activation may be a more salient feature of early‐onset or early‐stage hypertension [19–21]. In contrast, for individuals aged 45 and over, BMI was the dominant and consistent associate across nearly all models. This transition aligns with life‐course epidemiological data, which indicate that the association between adiposity and hypertension intensifies with age, while the independent effect of age itself diminishes after adjusting for cardiometabolic factors [22, 23].

Our finding that IL‐17A was additionally significant in the model of the older cohort is insightful. It suggests that in the context of established adiposity, the IL‐17/Th17 axis, a pathway implicated in sterile inflammation and autoimmune disease, may be recruited as a key mediator. This aligns with experimental evidence showing that IL‐17 promotes sodium retention, endothelial dysfunction, and vascular stiffness, and that its production can be stimulated by adipokines. Thus, our data support a two‐phase model: an earlier phase potentially driven by interfacing adaptive immunity and vascular aging, superseded by a later phase where metabolic dysfunction becomes paramount, with specific inflammatory pathways, such as IL‐17, acting as critical effector mechanisms [24, 25].

### 4.2. Influence of HIV Status on the Cytokine–SBP Associations

In contrast to the age‐associated models, the HIV status comparisons showed that both cytokines maintained significant associations with SBP regardless of HIV serostatus, with effect sizes that were remarkably consistent between the HIV + subgroup and the combined cohort. For IFN‐γ, the effect was nearly identical (*β* ≈ 0.00103–0.00104), while IL‐17A showed a slightly stronger effect in the HIV + subgroup (*β* = 0.0029 vs. 0.0027). This consistency suggests that HIV status does not fundamentally modify these cytokine– relationships, though the heightened inflammatory milieu in chronic HIV may modestly amplify the IL‐17A effect.

These findings are in agreement with prior studies which have shown that cardiovascular risk in HIV‐infected individuals is mediated by a combination of traditional risk factors, chronic inflammation, and HIV‐related immune dysregulation [26–28]. While chronic HIV infection is associated with persistent immune activation and elevated levels of pro‐inflammatory cytokines, including IL‐17A and IFN‐γ, our data suggest that the relationship between these cytokines and BP operates independently of HIV serostatus [29, 30]. However, the existing literature on whether inflammatory pathways function similarly in HIV‐infected and HIV‐uninfected populations remains limited, highlighting the need for further investigation. However, the slightly enhanced IL‐17A effect observed in the HIV + subgroup may reflect the well‐documented Th17 skewing that occurs in chronic HIV infection, though this interpretation remains speculative and warrants further investigation [31, 32].

### 4.3. Strengths, Limitations, and Future Directions

The study’s strengths include its biologically grounded, a priori age stratification and its simultaneous evaluation of multiple immune axes, which allowed for the detection of these nonlinear, age‐dependent relationships. The consistent adjustment for HIV status is a worthy noting strength given the cohort’s composition, allowing for a clearer interpretation of immune–metabolic associations within a population experiencing chronic immune activation. However, several limitations must be acknowledged when contextualizing these findings within the broader literature. The cross‐sectional design precludes causal inference; we therefore cannot determine whether elevated IFN‐γ in younger individuals is pathogenic or a consequence of early vascular injury. Longitudinal studies tracking immune and metabolic markers alongside BP from young adulthood into midlife are needed to validate the proposed sequence of drivers. The study relies on single‐time‐point measurements of circulating cytokines in a cross‐sectional design. This is a limitation since transient fluctuations in cytokine levels may not fully reflect chronic inflammatory states relevant to BP regulation; acute infections, circadian rhythms, or recent physical activity at the time of sampling could influence measured concentrations and potentially obscure or exaggerate associations with BP. Furthermore, the high prevalence of HIV, while informative for this growing population, may limit generalizability to the wider public. HIV and its treatment are known to alter immune function and metabolism, potentially accelerating or modifying the age‐related pathways observed. Future research should aim to replicate this stratified approach in HIV‐negative cohorts and employ more direct measures of vascular inflammation. Additional limitations include the use of research‐use‐only ELISA kits which have no clinical validation. Future studies employing clinically validated assays or multiplex platforms with broader dynamic ranges would help confirm these findings. Furthermore, cytokine concentrations were measured using RUO ELISA kits (Elabscience Biotechnology Co., Ltd.). These kits are not clinically validated, and their intended use is for detecting relative differences between experimental groups rather than establishing absolute diagnostic thresholds. The lack of clinical validation and the inherent interplatform variability across commercial ELISA kits limit direct comparison of absolute values with studies using alternative assays. Nevertheless, nonparametric statistical tests used for group comparisons remain robust for evaluating relative differences across age strata. Finally, the reliance on single‐point plasma cytokine measurements, as discussed, is a significant constraint. The field is increasingly moving toward profiling tissue‐resident immune cells, assessing cellular functional states, and using advanced imaging to visualize vascular inflammation, approaches that would provide a more direct test of the compartmentalized signaling model implied by our results.

## 5. Conclusion

This study tested the hypothesis that the relationship between specific immune signatures and BP is age‐dependent, with different inflammatory mediators being of relevance at different life stages. Our findings support this hypothesis. In younger adults (under 45 years), IFN‐γ emerged as a significant associate of SBP, consistent with a role for adaptive Th1‐driven immunity in early‐stage hypertension. In older adults (45 years and above), BMI superseded the inflammatory associations, thus supporting our hypothesis that metabolic factors become the dominant drivers of BP later in life. Notably, IL‐17A was significant in the older cohort, suggesting that Th17‐related pathways may serve as effector mechanisms in the context of established adiposity.

Stratified analyses by HIV status revealed that these age‐stratified cytokine–BP relationships were consistent between PLWH and HIV‐negative participants, indicating that the observed pathophysiological succession operates independently of HIV serostatus.

Put together, these findings, therefore, advance a life‐course understanding of inflammation in BP regulation, highlighting a transition from immune‐driven mechanisms in early adulthood to metabolic dominance in later life. This age‐stratified perspective encourages mechanism‐based, age‐tailored approaches to hypertension prevention and treatment, and shows the medical community the importance of research designs that respect biological heterogeneity across the lifespan.

## Author Contributions

Conceptualization, methodology, and data curation were conducted by Lweendo Muchaili and Sepiso K. Masenga. Investigation was performed by Lweendo Muchaili. Formal analysis, validation, and visualization were carried out by Annet Kirabo, Benson M. Hamooya, and Sepiso K. Masenga. Project administration and supervision were managed by Sepiso K. Masenga. Writing–original draft was prepared by Lweendo Muchaili. Writing–review and editing was completed by Elletina Nchimunya Sinamwenda, Joreen P. Povia, and Annet Kirabo. Funding acquisition was secured by Sepiso K. Masenga and Annet Kirabo.

## Funding

This work was supported by the Fogarty International Center and the National Institute of Diabetes and Digestive and Kidney Diseases of the National Institutes of Health under grant R21TW012635 (Sepiso K. Masenga and Annet Kirabo), and by grants R01HL147818 and R01HL144941 (Annet Kirabo). Additional support was provided by the American Heart Association, Award Number 24IVPHA1297559.

## Conflicts of Interest

The authors declare no conflicts of interest.

## Data Availability

The data that support the findings of this study are available from the corresponding author upon reasonable request.
